# AQ4N: a new approach to hypoxia-activated cancer chemotherapy

**DOI:** 10.1054/bjoc.2000.1564

**Published:** 2000-12

**Authors:** L H Patterson, S R McKeown

**Affiliations:** Department of Pharmaceutical and Biological Chemistry, School of Pharmacy, University of London, Brunswick Square, London, WC1N 1AX; School of Biomedical Sciences, University of Ulster at Jordanstown, BT37 0QB, Northern Ireland

**Keywords:** bioreductive, prodrug, topoisomerase II, in vivo, AQ4N, Tirapazamine

## Abstract

Preclinical studies demonstrate that in vivo AQ4N enhances the anti-tumour effects of radiation and chemotherapeutic agents with a dose-modifying factor of approximately 2.0. With careful scheduling no, or very little, additional normal tissue toxicity should be observed. AQ4N is a bioreductive prodrug of a potent, stable, reduction product which binds non-covalently to DNA, facilitating antitumour activity in both hypoxic and proximate oxic tumour cells. AQ4N is clearly different in both its mechanism of action and potential bystander effect compared to previously identified bioreductive drugs. In particular AQ4N is the only bioreductive prodrug topoisomerase II inhibitor to enter clinical trials. Targeting this enzyme, which is crucial to cell division, may help sensitize tumours to repeated (fractionated) courses of radiotherapy. This is because in principle, the bioreduction product of AQ4N can inhibit the topoisomerase activity of hypoxic cells as they attempt to re-enter the cell cycle. © 2000 Cancer Research Campaign http://www.bjcancer.com


*British Journal of Cancer* (2000) **83**(12), 1589–1593
© 2000 Cancer Research Campaign
doi: 10.1054/ bjoc.2000.1564, available online at http://www.idealibrary.com on IDEAL®

http://www.bjcancer.com

Mini-review

### AQ4N: a new approach to hypoxia-activated cancer chemotherapy

**LH Patterson[1] and SR McKeown[2]**

[1]Department of Pharmaceutical and Biological Chemistry, School of Pharmacy, University of London, Brunswick Square, London WC1N 1AX,
[2]School of Biomedical Sciences, University of Ulster at Jordanstown, Northern Ireland BT37 0QB

**Summary** Preclinical studies demonstrate that in vivo AQ4N enhances the anti-tumour effects of radiation and chemotherapeutic agents with a dose-modifying factor of approximately 2.0. With careful scheduling no, or very little, additional normal tissue toxicity should be observed. AQ4N is a bioreductive prodrug of a potent, stable, reduction product which binds non-covalently to DNA, facilitating antitumour activity in both hypoxic and proximate oxic tumour cells. AQ4N is clearly different in both its mechanism of action and potential bystander effect compared to previously identified bioreductive drugs. In particular AQ4N is the only bioreductive prodrug topoisomerase II inhibitor to enter clinical trials. Targeting this enzyme, which is crucial to cell division, may help sensitize tumours to repeated (fractionated) courses of radiotherapy. This is because in principle, the bioreduction product of AQ4N can inhibit the topoisomerase activity of hypoxic cells as they attempt to re-enter the cell cycle. © 2000 Cancer Research Campaign http://www.bjcancer.com

**Keywords**: bioreductive; prodrug; topoisomerase II; in vivo; AQ4N; Tirapazamine

### TUMOUR HYPOXIA AND BIOREDUCTIVE DRUGS

The development of a blood supply to provide oxygen and nutrients is essential for growth in a solid tumour. The chaotic and/or incomplete nature of the developing tumour vasculature results in disrupted delivery of oxygen creating subpopulations of cells which are either acutely or chronically hypoxic. Cycles of acute hypoxia/normoxia occur close to blood vessels due to intermittent vascular collapse. Cells distant (~70–150 microns) from a feeder vessel will become chronically hypoxic. These hypoxic cells are a significant problem in the control of solid tumours since they are 2–3 times more resistant to radiotherapy and are less sensitive to most chemotherapeutic agents (Brown, 2000). In addition, absence of oxygen promotes a stress response which may favour development of a more malignant phenotype (Graeber et al, 1996).

Efforts to eradicate hypoxic cells have led to the preclinical development of a number of bioreductive drugs. These are predominantly based on nitro or quinone functionality that can be reduced to covalent modifiers of DNA in hypoxic cells (reviewed in Patterson and Raleigh, 1998). Since hypoxia is found in tumours this provides for targeting of chemotherapy. Tirapazamine, a benzotriazene-di-N-oxide, was the first agent, not based on a nitro or quinone functionality, to exhibit a significant increase in cytotoxicity under hypoxic conditions. However Tirapazamine does exhibit some toxicity to oxic cells probably via a reactive oxygen species generated from reductase mediated redox cycling (Saunders et al, 2000). The recent success of Tirapazamine in clinical trials has championed the development of bioreductive drugs beyond preclinical rationalization (Brown, 1999).

*Received 9 October 2000*
*Accepted 19 October 2000*

*Correspondence to:* LH Patterson

Ideally a bioreductive prodrug should show minimal toxicity in oxic cells. In hypoxic cells it should be converted by an enzymic process, inhibited by oxygen, to a stable persistent cytotoxin. This would ensure that the prodrug, once reduced, will maintain activity should oxygen reperfusion occur. The product should bind non-covalently to DNA with an affinity high enough to produce cytotoxicity and low enough to allow slow diffusion and subsequent cytotoxicity in proximate tumour cells irrespective of their oxygen levels. AQ4N is an N-oxide of a DNA affinic agent that was rationalized to fulfil such requirements (Patterson, 1993).

### PHYSICAL PROPERTIES AND ASSAY OF AQ4N

AQ4N is the di-N-oxide of 1,4-bis[{2-(dimethylaminoethyl}-amino]5,8-dihydroxyanthracene-9,10-dione (AQ4), analkylamino-anthraquinone (see Figure 1). No detectable DNA binding could be shown in studies to determine the potential of AQ4N as a prodrug of a DNA-directed cytotoxic. In contrast its reduction product, AQ4, has a high DNA affinity constant which stabilizes the DNA double helix as measured by a large increase in DNA melting temperature. DNA binding of the AQ4 chromophore is greatly facilitated by the $\delta^+$ nature of the alkylamino side chains in the protonated form that allows for a critical electrostatic interaction with phosphates of the DNA backbone. In contrast, the $\delta^-$ partial charge on the N-oxide functionality of AQ4N makes

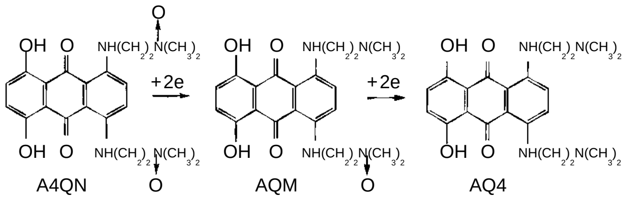

**Figure 1** AQ4N and its reduction metabolites such an interaction impossible; indeed, the electrostatic interaction with the DNA phosphates is repulsive. Hence AQ4N does not undergo a stable interaction with DNA.

The physical properties of AQ4N are considerably different to the active metabolite, AQ4. As an N-oxide of an aliphatic amine, AQ4N has salt-like properties which confer high aqueous solubility (260 mg $ml^{-1}$). This facilitates greatly the handling and formulation of AQ4N which has been routinely administered in water in all preclinical studies in vitro and in vivo. To assist in the large scale preparation for preclinical toxicology and clinical evaluation, AQ4N has been prepared as the dihydrochloride (Lee and Denny, 1999). The AQ4N.2HCl addition salt is possible because the N-oxide functionality retains basicity although considerably less so than AQ4. AQ4N.2HCl dissociates in aqueous solution and is indistinguishable from AQ4N. Since AQ4N is thermolabile at autoclave temperature (120°C) it cannot be heat sterilized. Instead it is formulated with phosphate salts as a filter sterilized, lyophilized powder which upon reconstitution with water provides a 40 mg $ml^{-1}$, pH 7.0 injection buffered solution. Studies have shown that aqueous solutions of AQ4N are stable at 4°C (for 28 days). Hence refrigeration is the preferred method of storage following reconstitution.

AQ4N is reduced to AQ4 through an obligate mono-N-oxide intermediate (AQM) and the final reduction product AQ4 (see Figure 1). To facilitate metabolism and pharmacokinetic studies a reverse phase HPLC method of analysis has been developed (Swaine et al, 2000). AQ4N is both hydrophilic and electrically neutral whilst AQ4 is extremely hydrophobic but cationic at acid pH. AQM retains cationic charge but is less hydrophobic. Detection is maximal at 242 nm although 267 nm, 612 nm and 662 nm can also be used consistent with the intense blue colour of AQ4N. AQ4N and AQ4 can also be visualized using flow cytometry and laser-scanning confocal fluorescence microscopy (Smith et al, 1997a). This is possible because both agents have intrinsic fluorescence in the far-red (650–800 nm) when illuminated with a Krypton laser (excitation at 647 nm or 514 nm).

### TUMOUR CELL AND TOPOISOMERASE II TARGETING BY AQ4N

When AQ4N (NSC 673504) was tested, in oxic conditions, against the National Cancer Institute (USA) panel of 60 tumour cell lines little or no cytotoxicity was observed ($IC_{50}$ >100 μM). In other studies, where reduction was facilitated, potent cytotoxicity was observed in the nM range (Patterson, 1993; Wilson et al, 1996; Smith et al, 1997b). In vitro the differential activity of AQ4N, to its active metabolite AQ4, is considerably greater than any other N-oxide described including DACA-N-oxide and nitracrine N-oxide (Wilson et al, 1996). Decreasing the cell culture medium to pH 6.5 had little effect on the cytotoxicity of AQ4 suggesting that its activity will be retained even if the hypoxic target cells are in regions of low extracellular pH.

Cellular uptake and subcellular distribution of AQ4N and AQ4 in single cells has been studied using flow cytometry and laser-scanning confocal fluorescence microscopy. These studies show cytoplasmic sequestration but not nuclear fluorescence of AQ4N. In contrast AQ4 is shown to be sequestered in the cytoplasm, nuclear membrane and intranuclear compartment. Following a 2 h incubation in drug-free medium as little as 1% of AQ4N was retained in the cells. In contrast up to 26% of AQ4 was retained in cells demonstrating the persistence of the cytotoxic metabolite (Smith, 1997a). This also demonstrates that the majority of AQ4, once formed is available to permeate into proximate tumour cells possibly affording a 'bystander' effect.

Topoisomerase II targeting by the DNA affinic AQ4 has been well documented (Patterson, 1993; Smith et al, 1997b). In contrast studies have shown that AQ4N was not able to inhibit decatenation of (kinetoplast) DNA by nuclear extract containing topoisomerase II (Patterson, 1993; Smith et al, 1997b). AQ4N showed no topoisomerase II trapping in cells with low (MRC5-V1) or elevated (AT5BIVA) topoisomerase II alpha expression as measured using $K^+$ SDS revealed DNA-protein cross-linking. Importantly, the cytotoxic reduction product, AQ4, showed higher total DNA crosslinking and more persistent enzyme trapping in the topoisomerase II overexpressing cell line. Consistent with this, the cytotoxicity of AQ4 was enhanced 21-fold in the topoisomerase II overexpressing cell line. The resistance of non-cycling cells to AQ4N and AQ4, as shown by a decreased cytotoxicity to plateau phase cells, is consistent with topoisomerase II poisoning as the major mechanism of action (Wilson et al, 1996). This may offer AQ4N a therapeutic advantage since it will limit the toxicity experienced by normal tissue which is predominantly composed of non-dividing cells. This is in addition to the fact that very limited metabolism of AQ4N should occur in normal tissues since they are normally adequately oxygenated.

### METABOLIC ACTIVATION OF AQ4N

Although it was rationalized that AQ4N would undergo bioreduction in hypoxic cells, early preclinical development of AQ4N was confounded by the lack of activity of AQ4N in several cell lines incubated under hypoxic conditions. Reduction of AQ4N to an in vitro cytotoxic product was only shown when liver microsomal fractions, known to be rich in cytochrome P450, were added to cultured cells. An over 100-fold increase in cytotoxicity was obtained when cells were incubated under hypoxic conditions with NADPH supplemented microsomes (Patterson, 1993). However, AQ4N is not activated by NAD(P)H-dependent cytochrome P450 reductase per se (Patterson et al, 1999), although this enzyme is well characterized as the reductase involved in nitro and quinone bioreductive drug activation and as a contributor to Tirapazamine activation (Patterson et al, 1998; Saunders et al, 2000). In the development of bioreductive drugs, the lack of activation of AQ4N by CPR is unique and is an advance on conventional bioreductives. This is because AQ4N, by comparison with other 1,4-disubstituted alkylaminoanthraquinones does not undergo cytochrome P450 reductase driven redox cycling and reactive oxygen formation (Fisher and Patterson, 1992). In contrast nitro and quinone based bioreductives as well as Tirapazamine all generate reactive oxygen under oxic conditions. Subsequently the lack of AQ4N metabolism in vitro was explained by the well recognized down-regulation of cytochrome P450 (CYP) expression in cell culture conditions. Indeed it was shown that excised tumour cells had the ability to metabolize AQ4N if exposed to hypoxic conditions and drug immediately on removal from a mouse; this activity was lost within 24 hours (Hejmadi et al, 1996).

Several studies have now addressed the nature of the CYP isoforms involved in AQ4N reduction. In humans the metabolism of AQ4N correlates significantly with the CYP3A subfamily of enzymes as measured using phenotyped liver microsomes as a mixed source of CYP (Raleigh et al, 1998). Using commercially

 © 2000 Cancer Research Campaign available recombinant CYPs (so-called supersomes), CYP1A1 and 2B6 are amongst the most efficient isoforms responsible for AQ4N reduction (unpublished data). In any given human tumour the full complement of CYPs expressed is not known. AQ4N reduction in a particular tumour is likely to be dependent on the presence of CYP1A, 2B6 and 3A and/or other as yet unidentified CYP isoforms. In the mouse CYP3A appears to play a role in AQ4N reduction consistent with some human studies (Patterson et al, 2000). However in the rat, inhibitor and induction studies show that CYP2B and 2E but not 3A are involved in AQ4N reduction (Raleigh et al, 1999) suggesting caution when interpreting CYP profiles is warranted. The specific mechanism of AQ4N reduction by CYPs is not known but given the requirement for NAD(P)H, native enzyme preparations and its inhibition by oxygen and carbon monoxide, it is likely that N-oxide interaction with the CYP haem-centred active site is required to facilitate nitrogen–oxygen bond cleavage. Essentially, reduction of the two N-oxide functionalities of AQ4N is likely to involve an oxygen atom transfer from the N-oxide side chains. This process is air sensitive, presumably because oxygen can out-compete AQ4N for haem binding.

There is some evidence that haem containing systems other than CYPs can mediate AQ4N reduction. A preliminary study has shown that recombinant nitric oxide synthase (NOS) reduces AQ4N (Raleigh, 1998). NOS is a homodimeric protein comprising a carboxy-terminal reductase domain resembling cytochrome P450 reductase and a P450-like N-terminal domain. Other chemotherapeutic agents including tirapazamine are substrates for several isoforms of NOS (Garner et al, 1999) and NOS is shown to be highly expressed in tumours (Thomsen et al, 1994, 1995).

### AQ4N REDUCTION IN TUMOURS

AQ4N is reduced to AQ4 in renal cell carcinoma microsomes (Patterson et al, 1999) which were also shown histochemically to express CYP (Murray et al, 1999). The carcinomas did not contain normal renal cells as monitored by histological examination. Colon adenocarcinoma microsomes also reduced AQ4N to its active metabolite (Patterson and Raleigh, unpublished results). The tumour expression of appropriate reductases is an important determinant in the potential of AQ4N activation to a cytotoxic metabolite. With this in mind, AQ4N-based prodrug therapy is likely to be an effective treatment regimen for a large number of patients with solid tumours since several drug metabolizing CYP isoforms have been detected in a broad spectrum of human cancers (reviewed in Patterson et al, 1999).

### AQ4N: DEFINING A MAXIMUM TOLERATED DOSE IN VIVO

It was observed that normoxic mice tolerated single doses up to 320 mg kg$^{-1}$ in the hypobaric chamber experiments (McKeown et al, 1995). In two studies over 400 mg kg$^{-1}$ was tolerated (Wilson et al, 1996, Bibby, personal communication); this has since been confirmed by preclinical toxicity trials carried out by BIBRA (personal communication). However the drug activity shows only a weak dependence on dose and between 100–200 mg kg$^{-1}$ an almost maximal anti-tumour effect is observed in growth delay of T50/80 tumour (McKeown et al, 1996). A similar maximal measurable effective limit is found at 200 mg kg$^{-1}$ in the SCCVII tumour using a clonogenic assay (Patterson et al, 2000).

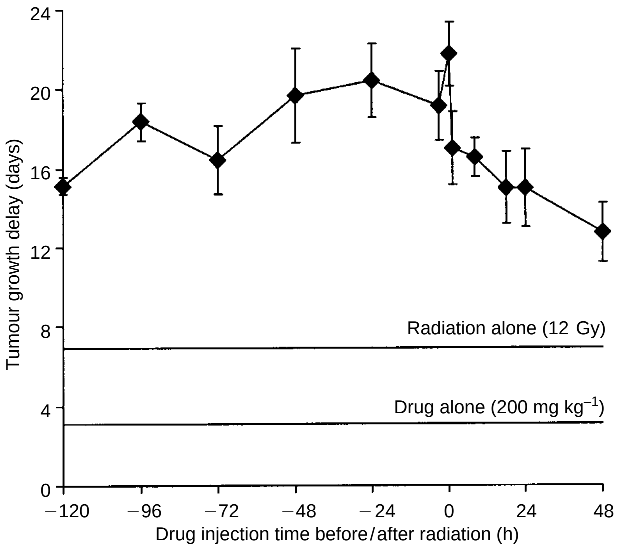

**Figure 2** Tumour growth delay when AQ4N was administered before or after a single dose of radiation (12 Gy). AQ4N (200 mg kg$^{-1}$) was administered at a range of times up to 120 h before and 48 h after a single dose of X-irradiation (12 Gy). Tumour growth delay (mean ± S.E.) (6–18 animals per group) is plotted against time of administration. Bars show the tumour growth delay obtained for AQ4N and radiation administered alone. The results are the pooled data from three experiments (McKeown et al, 1995)

### AQ4N AND HYPOXIA IN VIVO

The first demonstration that AQ4N had potential in vivo as a hypoxic cell cytotoxin was shown using a hypobaric chamber (McKeown et al, 1995). Previously this model system was used to show that the anti-tumour efficacy of Tirapazamine was significantly enhanced when mice were placed in a chamber at 0.5 atm (i.e. 10% oxygen) for 24 h following a single dose of a drug (McAleer et al, 1992). In the hypoxic chamber systemic toxicity was only minimally enhanced for AQ4N, whereas it was appreciable for Tirapazamine. This suggested that AQ4N had a minimal increase in metabolism in systemic tissues when the oxygen tension was lowered. In contrast, Tirapazamine is metabolized at all oxygen tensions and its bioreductive potential relates to the increased metabolism with low oxygen levels (Koch, 1993). Further support of the critical role of hypoxia in enhancing the anti-tumour efficacy of AQ4N was shown using in vivo experiments to combine AQ4N with a range of methods for inducing tumour hypoxia e.g. combination with the pharmacological vascular ‘steal’ agent hydralazine (Patterson et al, 2000) or dimethylxanthenone acetic acid (Wilson et al, 1996) or by vascular occlusion using clamping (Patterson et al, 2000); all showed enhanced cytotoxicity of AQ4N.

### AQ4N IN COMBINATION WITH RADIATION OR CHEMOTHERAPEUTIC AGENTS IN VIVO

AQ4N has a relatively weak antitumour activity per se. When combined with radiation in vivo a substantial enhancement of anti-tumour efficacy is observed with a dose modification factor of about two. This enhancement is found irrespective of whether AQ4N is administered before or after the radiation exposure providing strong evidence that this agent does act as a bioreductive cytotoxin (Figure 2; McKeown et al, 1995, 1996; Patterson et al,

© 2000 Cancer Research Campaign

**Table 1** Comparison of AQ4N with other clinically used bioreductive agents

| Agent | Chemical class | Mechanism of action | Activating enzyme | Active metabolite |
|---|---|---|---|---|
| AQ4N | Alkylaminoanthraquinone-N-oxide<br>N-oxide | DNA affinic<br>Topoisomerase II<br>inhibitor | Haemoproteins<br>e.g. CYP's, NOS | AQ4, stable<br>DNA affinic agent<br>Oxygen insensitive |
| Tirapazamine | Benzotriazene<br>di-N-oxide | DNA double<br>strand breaks | Flavoproteins<br>e.g. CPR,<br>nuclear reductases | Reactive nitroxyl<br>free radical,<br>Redox cycles in air |
| Mitomycin C/<br>Porfiromycin | Mitosene<br>(Indoloquinone) | DNA cross<br>linking | Flavoproteins<br>e.g. CPR, DTD | Alkylating species<br>Redox cycles in air |

CYP = cytochrome P450; NOS = nitric oxide synthase; CPR = cytochrome P450 reductase; DTD = DT diaphorase.

2000). Further evidence of the bioreductive nature of AQ4N was shown using the MDAH-Mca-4 tumour in vivo; the anti-tumour effect was enhanced with AQ4N when administered before or after radiation (Wilson et al, 1996). Treatment of tumour-bearing mice with AQ4N or radiation or a combination of both showed distinct patterns of DNA damage. This was measured by the 'Comet' assay which was performed on cells isolated from excised tumours subsequent to treatment (Hejmadi et al, 1996). Tumours treated with AQ4N alone showed little evidence of DNA damage whereas radiation alone showed immediate damage which was repaired typically after 24 h. Combination of radiation with AQ4N showed that the initial damage was repaired but a second prolonged peak of DNA damage was observed. We speculate that the prolonged damage is a result of formation of AQ4 and concomitant inhibition of topoisomerase II in hypoxic cells forced back into cycle as a consequence of ablation of the surrounding oxic cell fraction.

McKeown et al (1995) also showed that not only was there an enhancement of radiation cell kill but also the time at which the drug was administered which was not critical. A maximal effect was elicited when AQ4N was given up to 4 days before radiation. Administration of AQ4N after radiation also gave a maximal effect for at least 6 hours supporting the bioreductive nature of the interaction (Figure 2). The interaction time fell off faster when AQ4N was administered after radiation probably because reoxygenation will reduce the number of cells sensitive to all bioreductive drugs, including AQ4N. In a study of a faster-growing tumour (SCCVII) maximal interaction was seen over a 16 hour period irrespective of whether the drug was given before or after radiation (Patterson et al, 2000). The shorter time of interaction may well be explained by the faster growth rate of cells in the SCCVII compared to the T50/80 tumour. Essentially the production of AQ4 in hypoxic cells will compromise the cells in which it is generated for some considerable time due to the stable and DNA affinic nature of AQ4. The effect of AQ4 is likely to disappear in a faster-growing tumour (i.e. SCCVII) more quickly than a slow-growing tumour (e.g. T50/80) since new oxic cells will be produced more quickly. Overall the relatively long interaction time for both these tumour models (i.e. in the region of 1–4 days) highlights a potential benefit of AQ4N in the clinical setting since it will facilitate scheduling with radiotherapy or oxic cell cytotoxins (see below).

Following the success of the single dose radiation/AQ4N combinations the efficacy of two different fractionated drug/fractionated radiation combinations was investigated (McKeown et al, 1995, 1996). Although maximal anti-tumour effects were obtained when the drug was given daily there was an appreciable effect when AQ4N dosing was on a once- or twice-weekly basis. The results suggest that an appreciable effect has only a limited dependence on drug dose or fractionation regimen. Enhancement of the anti-tumour efficacy with multiple dosing may be explained by the increased number of acutely hypoxic cells which will be killed in a fractionated schedule ($5 \times 40$ mg kg$^{-1}$) as compared to a single dose (200 mg kg$^{-1}$). The results highlight that flexibility of AQ4N scheduling should be possible.

Combination of AQ4N with thiotepa, cyclophosphamide and cisplatin has also shown enhanced anti-tumour responses in different tumour models in vivo (Patterson et al, 2000). With cyclophosphamide the enhancement has a dose modification of approximately 2 (Friery et al, 2000) and is of the same order as that obtained in the single dose radiation experiments.

### DOES AQ4N PROVIDE A THERAPEUTIC GAIN?

There is now good evidence that AQ4N has significant anti-tumour efficacy in vivo when combined with a number of oxic cell cytotoxins (see above). For this to translate into clinical benefit AQ4N must provide a therapeutic gain. In support of this AQ4N alone showed no bone marrow toxicity when given with a 6 h interval in combination with cyclophosphamide or cisplatin (Friery et al, 2000; Gallagher et al, personal communication). AQ4N given 0.5 h prior to chemotherapy showed no, or only limited, enhancement. The effect of scheduling AQ4N and chemotherapy at intervals between 0.5–6 h has not been examined. Other normal tissue end-points have also been investigated. Radiation did not enhance the toxicity of AQ4N to the eccrine sweat glands of the mouse foot (McKeown et al, 1996). AQ4N was not toxic to the mouse retina whereas certain nitro-based bioreductive agents were significantly retino-toxic and even Tirapazamine displayed measurable toxicity (Lee and Wilson, 2000). AQ4N in combination with radiation has only minimal toxicity to mouse skin (McIntyre, personal communication).

### SUMMARY

AQ4N enhances the anti-tumour efficacy of radiation and standard chemotherapeutic agents with a dose-modifying factor of approximately 2.0. With careful scheduling no, or very little, additional normal tissue toxicity should be observed. AQ4N is a bioreductive prodrug of a potent, stable, reduction product which binds non-covalently to DNA, facilitating antitumour activity in both hypoxic and proximate oxic tumour cells. AQ4N is clearly different from previously identified bioreductive drugs (see Table 1). In particular AQ4N is the only bioreductive prodrug

 © 2000 Cancer Research Campaign topoisomerase II inhibitor to enter clinical trials. Targeting this enzyme, which is crucial to cell division, may help sensitize tumours to repeated (fractionated) courses of radiotherapy. This is because in principle, the bioreduction product of AQ4N can inhibit the topoisomerase activity of hypoxic cells as they attempt to re-enter the cell cycle.

### REFERENCES

Brown JM (1999) The hypoxic cell: a target for selective cancer therapy – 18th Bruce F. Caine Memorial Award Lecture. *Cancer Res* **59(23)**: 5863–5870

Brown JM (2000) Exploiting the hypoxic cancer cell: mechanisms and therapeutic strategies. *Mol Med Today* **6(4)**: 157–162

Fisher GR and Patterson LH (1992) Lack of involvement of reactive oxygen in the cytotoxicity of mitoxantrone, CI941 and ametantrone in MCF-7 cells: comparison with doxorubicin. *Cancer Chemother Pharmacol* **30(6)**: 451–458

Friery OP, Gallagher R, Murray MM, Hughes CM, Galligan ES, McIntyre IA, Patterson LH, Hirst DG and McKeown SR (2000) Enhancement of the anti-tumour effect of cyclophosphamide by the bioreductive drugs AQ4N and tirapazamine *Br J Cancer* **2(8)**: 1469–1473

Garner AP, Paine MJ, Rodriguez-Crespo I, Chinje EC, Ortiz De Montallano P, Stratford IJ, Tew DG and Wolf CR (1999) Nitric oxide synthases catalyse the activation of redox cycling and bioreductive anticancer agents. *Cancer Res* **59(8)**: 1929–1934

Graeber TG, Osmanian C, Jacks T, Housman DE, Koch CJ, Loke SW and Giaccia AJ (1996) Hypoxia-mediated selection of cells with diminished apoptotic potential in solid tumours. *Nature* **379(6560)**: 88–91

Hejmadi MV, McKeown SR, Friery OP, McIntyre IA, Patterson LH and Hirst DG (1996) DNA damage following combination of radiation with the bioreductive drug AQ4N: possible selective toxicity to oxic and hypoxic tumour cells *Br J Cancer* **73(4)**: 499–505

Koch J (1993) Unusual oxygen concentration-dependence of toxicity of SR-4233, a hypoxic cell cytotoxin. *Cancer Res* **53(17)**: 3992–3997

Lee HH and Denny WAA (1999) large-scale synthesis of the bioreductive drug 1,4-bis{[2-(dimethylamino)ethyl]amino}-5,8-dihydroxyanthracene-9,10-dione bis-N-oxide (AQ4N). *J Chem Soc Perkins Trans I* **19**: 2755–2758

Lee AE and Wilson WR (2000) Hypoxia-dependent retinal toxicity of bioreductive anticancer prodrugs in mice. *Toxicol Appl Pharmacol* **163(1)**: 50–59

McKeown SR, Hejmadi MV, McIntyre IA, McAleer JJ and Patterson LH (1995) AQ4N: an alkylaminoanthraquinone N-oxide showing bioreductive potential and positive interaction with radiation in vivo. *Br J Cancer* **72(1)**: 76–81

McKeown SR, Friery OP, McIntyre IA, Hejmadi MV, Patterson LH and Hirst DG (1996) Evidence for a therapeutic gain when AQ4N or tirapazamine is combined with radiation. *Br J Cancer Suppl* **27**: S39–42

Murray GI, McFadyen MCE, Mitchell RT, Cheung YL, Kerr AC and Melvin WT (1999) Cytochrome P450CYP3A in human renal cell cancer. *British Journal of Cancer* **79**: 1836–1842

Patterson LH (1993) Rationale for the use of aliphatic N-oxides of cytotoxic anthraquinones as prodrug DNA binding agents: a new class of bioreductive agent. *Cancer Metastasis Rev* **12(2)**: 119–134

Patterson LH and Raleigh SM (1998) Reductive metabolism: Its application to prodrug activation. In: NJ Gooderham (ed) *Drug Metabolism: Towards the Next Millennium*. IOS Press: Amsterdam, pp. 72–79

Patterson LH, Craven MR, Fisher GR and Teesdale-Spittle P (1994) Aliphatic amine N-oxides of DNA binding agents as bioreductive drugs. *Oncol Res* **6(10–11)**: 533–538

Patterson AV, Saunders MP, Chinje EC, Patterson LH & Stratford IJ (1998). Enzymology of tirapazamine metabolism: a review. *Anticancer Drug Des* **13**: 541–573

Patterson LH, McKeown SR, Robson T, Gallagher R, Raleigh SM and Orr S (1999) Antitumour prodrug development using cytochrome P450 (CYP) mediated activation. *Anticancer Drug Des* **14(6)**: 473–486

Patterson LH, McKeown SR, Ruparelia K, Double JA, Bibby MC, Cole S and Stratford IJ (2000) Enhancement of chemotherapy and radiotherapy of murine tumours by AQ4N, a bioreductively activated anti-tumour agent. *Br J Cancer* **82(12)**: 1984–1990

Raleigh SM (1998) Involvement of cytochrome P450 (CYP) and other haem associated enzymes in the bioreduction of AQ4N, an antitumour produrg. PhD thesis, De Montfort University, Leicester

Raleigh SM, Wanogho E, Burke MD, McKeown SR and Patterson LH (1998) Involvement of human cytochromes P450 (CYP) in the reductive metabolism of AQ4N, a hypoxia activated anthraquinone di-N-oxide prodrug. *Int J Radiat Oncol Biol Phys* **42(4)**: 763–767

Raleigh SM, Wanogho E, Burke MD and Patterson LH (1999) Rat cytochromes P450 (CYP) specifically contribute to the reductive bioactivation of AQ4N, an alkylaminoanthraquinone-di-N-oxide anticancer prodrug *Xenobiotica* **(11)**: 1115–1122

Saunders MP, Patterson AV, Chinje EC, Harris AL and Stratford IJ (2000) NADPH: cytochrome c(P450) reductase activates tirapazamine (SR4233) to restore hypoxic and oxic cytotoxicity in an aerobic resistant derivative of the A549 lung cancer cell line. *Br J Cancer* **82(3)**: 651–656

Smith PJ, Desnoyers R, Blunt N, Giles Y, Patterson LH and Watson JV (1997a) Flow cytometric analysis and confocal imaging of anticancer alkylaminoanthraquinones and their N-oxides in intact human cells using 647-nm krypton laser excitation. *Cytometry* **27(1)**: 43–53

Smith PJ, Blunt NJ, Desnoyers R, Giles Y and Patterson LH (1997b) DNA topoisomerase II-dependent cytotoxicity to alkylaminoanthraquinones and their N-oxides. *Cancer Chemother Pharmacol* **39(5)**: 455–461

Swaine DJ, Loadman PM, Bibby MC, Graham MA and Patterson LH (2000) High-performance liquid chromatographic analysis of AQ4N, an alkylaminoanthraquinone N-oxide. *J Chromatogr B Biomed Sci Appl.* **742(2)**: 239–245

Thomsen LL, Lawton FG, Knowles RG, Beesley JE, Riveros-Moreno V and Moncada S (1994) Nitric oxide synthase activity in human gynaecological cancer. *Cancer Res* **54(5)**: 1352–1354

Thomsen LL, Miles DW, Happerfield L, Bobrow LG, Knowles RG and Moncada S (1995) Nitric oxide synthase activity in human breast cancer. *Br J Cancer* **72(1)**: 41–44

Wilson WR, Denny WA, Pullen SM, Thompson KM, Li AE, Patterson LH and Lee HH (1996) Tertiary amine N-oxides as bioreductive drugs: DACA N-oxide, nitracrine N-oxide and AQ4N. *Br J Cancer Suppl* **27**: S43–47

© 2000 Cancer Research Campaign

